# Mouse models of Kras activation in gastric cancer

**DOI:** 10.1038/s12276-022-00882-1

**Published:** 2022-11-11

**Authors:** Yoonkyung Won, Eunyoung Choi

**Affiliations:** 1 Department of Surgery, Vanderbilt University Medical Center, Nashville, TN 37232 USA; 2 Epithelial Biology Center, Vanderbilt University Medical Center, Nashville, TN 37232 USA; 3 Department of Cell and Developmental Biology, Vanderbilt University, Nashville, TN 37232 USA

**Keywords:** Cancer models, Cancer models

## Abstract

Gastric cancer has one of the highest incidence rates and is one of the leading causes of cancer-related mortality worldwide. Sequential steps within the carcinogenic process are observed in gastric cancer as well as in pancreatic cancer and colorectal cancer. Kirsten rat sarcoma viral oncogene homolog (KRAS) is the most well-known oncogene and can be constitutively activated by somatic mutations in the gene locus. For over 2 decades, the functions of Kras activation in gastrointestinal (GI) cancers have been studied to elucidate its oncogenic roles during the carcinogenic process. Different approaches have been utilized to generate distinct in vivo models of GI cancer, and a number of mouse models have been established using Kras-inducible systems. In this review, we summarize the genetically engineered mouse models in which Kras is activated with cell-type and/or tissue-type specificity that are utilized for studying carcinogenic processes in gastric cancer as well as pancreatic cancer and colorectal cancer. We also provide a brief description of histological phenotypes and characteristics of those mouse models and the current limitations in the gastric cancer field to be investigated further.

## Gastrointestinal cancer: Progress in mouse models

Improvements to genetically engineered mouse models for studying a specific oncogene, a gene with the potential to cause cancer, in gastrointestinal (GI) cancers could help identify the precise cellular origins of these diseases. Mutations affecting the Kirsten rat sarcoma viral oncogene (*Kras*) are among the most common drivers of cancer. *Kras* activation varies in terms of incidence rates and roles across GI cancers. Eunyoung Choi and Yoonkyung Won at Vanderbilt University Medical Center in Nashville, USA, reviewed the current scope of genetically engineered mouse models for studying *Kras* in gastric, pancreatic, and colorectal cancers. Such models have contributed extensively to knowledge of alternate functions of Kras activation during carcinogenic process in different types of cell lineages or organs, and identification of molecular signatures underlying *Kras* activation.

## Introduction

The gastrointestinal (GI) tract, as a part of the digestive system, includes the esophagus, stomach, pancreas, small intestine and colon; the GI tract is where foods and liquids enter into the body and are digested and nutrients are absorbed^1^. GI cancers represent a substantial proportion of cancer incidence and mortality worldwide^2^. GI cancers develop in a sequential carcinogenic process through a series of preneoplastic lesions. In the stomach, intestinal-type gastric cancer is the most common cancer type and is associated with environmental factors, such as acute mucosal injury by toxic drugs and chronic inflammation caused by *Helicobacter pylori* infection^3,4^. Intestinal-type gastric cancer develops within these preneoplastic metaplastic lesions from normal mucosal changes through chronic gastritis with mucosal atrophy and a multistep process, which involves the progression of preneoplastic pyloric metaplasia and intestinal metaplasia (IM) to neoplastic dysplasia and adenocarcinoma. These sequential changes were first described as the Correa pathway by Pelayo Correa^5^. Pyloric metaplasia can initially arise following acid-secreting parietal cell atrophy through the transdifferentiation of zymogen granule-secreting chief cells into metaplastic cells, called spasmolytic polypeptide-expressing metaplasia (SPEM) cells, in response to mucosal injury^6–9^. While this initial process is potentially reversible, cell plasticity also permits the entry of metaplastic cells into carcinogenic transition, leading to the progression of reversible pyloric metaplasia to irreversible IM and neoplastic dysplasia^10,11^. This carcinogenic cascade is also observed in other GI tract cancers, such as esophageal, pancreatic and colorectal cancers^12–14^. In pancreatic carcinogenesis, several types of preneoplastic lesions have been identified, including pancreatic intraepithelial neoplasia (PanIN), intraductal papillary mucinous neoplasia (IPMN) and mucinous cystic neoplasia (MCN)^15^. PanINs are characterized by a stepwise acquisition of mutations in Kras and Trp53 genes from low-grade dysplasia (PanIN 1-2) to carcinoma in situ (PanIN 3)^16^. Colorectal cancer also develops from the progression of acquired or hereditary premalignant lesions^17,18^. Colorectal carcinogenesis progresses from hyperproliferative regions in the normal colonic mucosa designated as polyps into early and late adenoma and finally carcinoma^14,19^.

Mutations influencing members of the rat sarcoma viral oncogene family (RAS) genes (KRAS, NRAS, HRAS) are the most frequent genetic alterations in human cancers, accounting for approximately 30% of all tumors^20^. Ras proteins function as a simple binary ON–OFF molecular switch through the function of guanosine triphosphatase (GTPase), which controls cycles between an active guanosine triphosphate (GTP)-bound and inactive guanosine diphosphate (GDP)-bound state^21^. Kras is predominantly inactive and GDP-bound in quiescent cells, while it is active and GTP-bound in active cells where extracellular stimuli activate receptor tyrosine kinases (RTKs) and other cell surface receptors. In cancers, the Ras genes harbor missense mutations that encode single amino acid substitutions primarily at one of three mutational spots: glycine-12 (G12), glycine-13 (G13), or glutamine-61 (Q61). These mutations block GTPase-activating proteins (GAPs) from accessing GTP, and hydrolysis is prevented, resulting in persistent activation of the GTP-bound state^22^. In 1982, the Kras gene was the first oncogene identified in human cancer^23^ and one of the proto-oncogenes predominantly mutated in many GI cancers, including pancreatic, gastric, and colorectal cancer^24–27^. Because of various incidence rates and roles of Kras activation in different GI cancers, numerous studies have been performed to elucidate the oncogenic mechanisms of Kras in GI carcinogenesis^11,28–36^.

Experimental studies that induce mutations in animals can typically drive initiation processes in the development and promotion of the following stages. Many efforts have been made to investigate the detailed mechanisms of the carcinogenic process using genetically engineered mouse models (GEMMs). Therefore, GEMMs have led to enormous advancements in understanding the fundamentals of tumor initiation, development, and metastatic spread. In the cancer field, many groups have put research efforts into the generation of preclinical mouse models for GI cancer studies^37–41^. In particular, the generation of the LoxP-STOP-LoxP (LSL)-Kras^G12D^ mouse in 2001^42^ allowed the expression of the mutant Kras allele in specific cell types under Cre recombinase activity controlled by the endogenous transcriptional activity of the driver gene locus. Here, we summarize GEMMs that develop active Kras-induced GI carcinogenesis and have been commonly used as in vivo mouse models in the GI cancer field. We also discuss the distinct functional roles and different histological phenotypes of GEMMs in gastric, pancreatic cancer, and colorectal cancers.

## Kras activation in GI cancers

Several studies on the molecular profiling of gastric cancer have been performed to examine distinct molecular subtypes with clinical characteristics^43–48^. In particular, molecular characterization of gastric cancer cases by the TCGA project classified these cases into four distinct groups: Epstein‒Barr virus (EBV), microsatellite instability (MSI), genomically stable (GS), and chromosomal instability (CIN) subtypes. Among them, the CIN subtype is the most common subtype associated with intestinal-type histology and is characterized by genetic amplification/activation of the RTK/RAS signaling pathway and frequent Trp53 mutation^43^. Even though mutations of Kras are detected in approximately 10–15% of all gastric cancer cases, signatures for the activation and amplification of Kras are noted in at least 40% of human intestinal-type gastric cancers.

On the other hand, KRAS mutations are observed in approximately 90% of pancreatic cancer patients, and mutations in tumor suppressors such as CDKN2A/p16INK4A, Trp53, and SMAD4 are also common in pancreatic cancer. Of note, Kras mutations are an early oncogenic event in pancreatic carcinogenesis^49–53^. Colorectal cancer also develops through a series of germline or somatic mutations, which affect the homeostasis of oncogenes or tumor suppressors. A large proportion of somatic mutations have been identified in colorectal cancer, including mutations in Trp53, APC, KRAS, PIK3CA, SMAD4, FBXW7, and RNF43, which drive the progression of preneoplastic lesions to malignant colorectal cancer^54^.

## GEMMs of Kras activation in the pancreas and colon

Mouse models of pancreatic cancer have been extensively well developed and utilized for several decades (Table 1). The GEMMs of pancreatic cancer display phenotypes of all recognized features observed in human pancreatic cancer development and progression, from the preneoplastic PanIN to invasive adenocarcinoma, representing the major example of Kras activation in the gastrointestinal cancer field. The GEMMs for studying pancreatic cancer were mostly generated using the Pdx1-Cre/CreERT or Ptf1a(P48)-Cre driver mouse allele^32–34,55–60^. Pdx1 is not a pancreas tissue-specific gene and is observed in the pancreas and the distal part of the stomach and duodenum. However, the Pdx1-Cre/CreERT mouse allele is often used as a ductal cell driver model in the pancreas^32,34,55–58^. The Ptf1a(P48)-Cre mouse allele is a pancreas tissue-specific and acinar cell-specific driver model^32,34,59,60^. Although Kras activation is induced in either ductal cells or acinar cells, both mouse models develop the full range of PanIN lesions and rare invasive adenocarcinomas that are histologically similar to those present in human patients^32,33^. Additionally, Kras activation in acinar cells using the Mist1-CreERT2, proCPA1-CreERT2, or Ela-CreERT2 mouse alleles also shows PanIN development with focal cystic neoplasia^33,61^. On the other hand, concurrent Kras activation and Trp53 deletion or coactivation of KrasG12D and Trp53R172H in the pancreas displayed malignant progression of preneoplastic PanIN to neoplastic stages^33,34,62^. Therefore, Trp53 mutations might be required for neoplastic transition from preneoplastic PanIN induced by Kras activation during pancreatic cancer development.

**Table 1:** Genetically engineered mouse models of pancreatic cancer.

GEMM	References
Pdx1-Cre; LSL-Kras^G12D/+^	Hingorani et al., 2003
Ptf1a(P48)-Cre; LSL-Kras^G12D/+^	
Ela-CreERT2; LSL-Kras^G12D/+^	Habbe et al., 2008
Mist1-CreERT2; LSL-Kras^G12D/+^	
Pdx1-CreERT; LSL-Kras^G12D/+^	Gidekel Friedlander et al., 2009
Pdx1-CreERT; LSL-Kras^G12D/+^;Trp53^fl/fl^	
Pdx1-CreERT; LSL-Kras^G12D/+^;Ink4a/Arf^fl/fl^ ProCPA1-CreERT2; LSL-Kras^G12D/+^	
ProCPA1-CreERT2; LSL-Kras^G12D/+^;Trp53^fl/fl^	
ProCPA1-CreERT2; LSL-Kras^G12D/+^;Ink4a/Arf^fl/fl^	
Pdx1-Cre; LSL-Kras^G12D/+^;Ink4a/Arf^fl/fl^	Aguirre et al., 2003
Pdx1-Cre; LSL-Kras^G12D/+^;LSL-Tp53^R172H/+^	Hingorani et al., 2005
Pdx1-Cre; LSL-Kras^G12D/+^;Trp53^fl/fl^	Bardeesy et al., 2006
Pdx1-Cre; LSL-Kras^G12D/+^; Ink4a/Arf^fl/+ or fl/fl^	
Pdx1-Cre; LSL-Kras^G12D/+^;Pten^fl/+ or fl/fl^	Ying et al., 2011
Pdx1-Cre; LSL-Kras^G12D/+^;Lkb1^fl/+^	Morton et al., 2010
Pdx1-Cre; LSL-Kras^G12D/+^;p21Cip1^+/-^	
Pdx1-Cre; LSL-Kras^G12D/+^;Brca2^fl/fl^	Skoulidis et al., 2010
Ptf1a(P48)-Cre; LSL-Kras^G12D/+^;TGFbRII^fl/fl^	Ijichi et al., 2006
Ptf1a(P48)-Cre; LSL-Kras^G12D/+^;Smad4^fl/+ or fl/fl^;LSL-Tp53^R172H/+^	Whittle et al., 2015

In the colon, the initial step in carcinogenesis is proposed to be the loss of the tumor suppressor gene Apc. The inactivation of Apc induces the β-catenin stabilization and translocation to the nucleus. Kras activation is an event during tumor progression, and the mutation is observed in approximately 40–50% of human colorectal cancer patients. However, the role of Apc loss of heterozygosity, rather than Kras activation, as a key mutation initiating the carcinogenic process in the colon is highlighted in several mouse models^63–67^. Among the inducible driver mouse alleles for studying colorectal cancer (Table 2), the Villin-Cre driver system, either constitutive expression of Cre (Villin-Cre) or tamoxifen-inducible expression of Cre (Villin-CreERT2), is the most common tool to restrict Cre recombinase activity to intestinal epithelial cells in both small intestine and colon^68^. Kras activation using the Villin-Cre/CreERT alleles was found to be insufficient to lead to the full process of colorectal carcinogenesis. However, carcinogenesis was promoted, and tumors developed with additional oncogenic gene mutations or treatment with chemicals, such as azoxymethane (AOM), in the colon^35,69–72^. Another driver of site-specific Cre expression is the fatty acid binding protein liver Cre transgene (Fabpl-Cre)^67,73^. While Villin-Cre is activated in the intestinal epithelial cells of the entire region of both the small intestine and the colon, Fabpl-Cre expression is detected in the distal small intestine, cecum, and colon^64,73,74^. The Ah-Cre driver under the control of the Cyp1A promotor is also used for colon cancer models, but the Cyp1A gene is also expressed in the liver, pancreas, and esophageal epithelium^66,75^. Moreover, several studies have focused on the function of intestinal stem cells using Lgr5- or Lrig1-CreERT2 mice^76–78^. Lgr5-CreERT2 mice with Apc deletion were found to exhibit adenoma formation^76^, but mice with Lrig1-CreERT2 with Apc deletion developed duodenal adenoma and superficially invasive adenocarcinoma^77,78^. However, few investigations have examined the oncogenic roles of Kras activation alone in depth using intestinal epithelial cell driver alleles, suggesting that Kras activation might be the second event in colorectal carcinogenesis. Therefore, it is not yet clear whether a single oncogenic activation of Kras is sufficient to develop adenocarcinoma in GI organs, and orchestrated induction of several oncogenes may be required for the full completion of carcinogenesis.

**Table 2:** Genetically engineered mouse models of colorectal cancer.

GEMM	References
Villin-Cre; LSL-Kras^G12D/+;^ +AOM	Calcagno et al., 2008
Villin-Cre; LSL-Kras^G12D/+;^Tgfbr2^E2fl/E2fl^	Trobridge et al., 2009
Villin-Cre; LSL-Kras^G12D/+^	Bennecke et al., 2010
Villin-Cre; Kras^G12Dint^	
Villin-Cre; Kras^G12Dint^;Ink4a/Arf^−/−^	
Villin-CreERT; LSL-Kras^G12D/+^;Apc^fl/+^;Pten^fl/fl^	Davies et al., 2014
AhCre; LSL-Kras^V12/+^	Sansom et al., 2006
AhCre; LSL-Kras^V12/+^, Apc^fl/+ or fl/fl^	
Fapbl-Cre; LSL-Kras^G12D/+^;Apc^2lox14/+^	Haigis et al., 2008
Fabpl-Cre; LSL-Kras^G12D/+^	Tuveson et al., 2004
Ah-Cre;Apc^fl/fl^	Barker et al., 2009
Lgr5-CreERT2;Apc^fl/fl^	Powell et al., 2012
Lrig1-CreERT2;Apc^fl/+^	Powell et al., 2014

## GEMMs of Kras activation in the stomach

Gastric glands consist of multiple types of gastric cell lineages, such as mucin-secreting surface/foveolar cells or mucus neck cells, acid-secreting parietal cells, zymogen granule-secreting chief cells, and proliferating isthmal progenitor cells as well as endocrine cells and tuft cells^79^. In recent decades, a number of driver mouse models that induce Cre recombinase in gastric cell lineages have been generated, and many studies have been conducted to elucidate the molecular mechanisms of Kras activation in gastric carcinogenesis by generating Cre recombinase-inducible Kras transgenic mouse alleles (Table 3). The first experimental insight into the role of oncogenic Kras in the gastric epithelium was from a study using the keratin 19 (K19) promoter to induce Kras^V12^ directly in the gastric epithelium by the Rustgi group^80^. K19 transcription was observed in the isthmal progenitor cell zone, and Kras^V12^ induction by K19 promoter activity resulted in mucous neck cell hyperplasia and parietal cell loss. A further study from the Wang group showed that K19-Kras^V12^ transgenic mice with *Helicobacter felis* bacterial infection showed parietal cell loss, metaplasia, and dysplasia, and adenocarcinoma was found in 38% of the mice by 16 months of age, concomitant with an early upregulation of CXCL1 and the recruitment of bone marrow-derived cells and fibroblasts^81^. In addition, a conditional LSL-Kras^G12D^ mouse allele was introduced to induce Kras activation by the generation of a new inducible Cre driver mouse allele using the K19 gene locus^82^. The phenotype of this mouse allele included prominent foveolar hyperplasia, metaplasia, and adenomas in the stomach as well as in the oral cavity, colon, and lungs. Notably, only small numbers of mucinous metaplasias with characteristics of early-stage PanINs developed in the pancreas.

**Table 3:** Genetically engineered mouse models of gastric cancer.

GEMM	References
K19-Kras-V12 TG	Brembeck et al., 2003
K19-Kras-V12 TG	Okumura et al., 2010
K19-CreERT; LSL-Kras^G12D/+^	Ray et al., 2011
Ubc9 Cre-ERT2; LSL-Kras^G12D/+^	Matkar et al., 2011
Mist1-CreERT2; LSL-Kras^G12D/+^	Choi et al., 2016
eR1-CreERT2; LSL-Kras^G12D/+^	Matsuo et al., 2017
Atp4b-Cre; LSL-Kras^G12D/+^; Cdh1^fl/fl^; Trp53^fl/fl^	Till et al., 2017
Tff1-Cre; LSL-Kras^G12D/+^	Kinoshita et al., 2019
Tff1-Cre; LSL-Kras^G12D/+^;Pten^fl/fl^	
Anxa10-CreERT2; LSL-Kras^G12D/+^;LSL-Tp53^R172H/+^;Smad4^fl/fl^	Seidlitz et al., 2019
Anxa10-CreERT2; LSL-Kras^G12D/+^;Cdh1^fl/fl^;Smad4^fl/fl^	
Anxa10-CreERT2; LSL-Kras^G12D/+^;Cdh1^fl/fl^;Apc^fl/fl^	
Lrig1-CreERT2; LSL-Kras^G12D/+^	Choi et al., 2019
Iqgap3-CreERT2; LSL-Kras^G12D/+^	Matsuo et al., 2021
Pgc-CreERT2; LSL-Kras^G12D/+^	Douchi et al., 2021
Pgc-CreERT2; LSL-Kras^G12D/+^; Apc^fl/fl^	
Pgc-CreERT2; LSL-Kras^G12D/+^; Apc^fl/fl^;Trp53^fl/+ or fl/fl^	
Cldn18-CreERT2; LSL-Kras^G12D/+^	Fatehullah et al., 2021
Cldn18-CreERT2; LSL-Kras^G12D/+^;Apc^fl/fl^	
Cldn18-CreERT2; LSL-Kras^G12D/+^;Trp53^fl/fl^	
Cldn18-CreERT2; LSL-Kras^G12D/+^;Apc ^fl/+ or fl/fl^;Trp53^fl/fl^	

The ubiquitin-conjugating enzyme 9 (Ubc9) gene is a small ubiquitin-like protein modifier (SUMO)-conjugating protein, and various cell types across tissues express this protein^83^. The Hua group crossed the Ubc9-CreERT driver mouse allele with LSL-Kras^G12D^ mice to examine the effect of Kras activation in many organs, including GI tract organs^84^. The Ubc9-CreERT;LSL-Kras^G12D^ mice started dying approximately 2 weeks after Kras induction but did not show any obvious tumor formation in the GI organs, such as pancreas, liver, small intestine, and colon, nor in the lungs and kidneys, except for one mouse showing oral papilloma. In the stomach tissue, the mice showed rapid changes and dramatic effects in both the forestomach and glandular stomach, severe inflammation, hyperplasia, and metaplasia were observed in the stomach without neoplasia. These results suggest that, among all the tissues in which Kras is activated, the stomach appears to be particularly susceptible to Kras activation at early time points; therefore, Kras activation may have crucial roles in the initiation of the carcinogenic process in the stomach. However, although Kras^G12D^ GEMMs using these driver mouse alleles described above do display critical features of gastric carcinogenesis, they do not display all of the critical features seen in human patients with intestinal-type gastric cancer, and chronic inflammation by *Helicobacter* bacterial infection might be required to promote preneoplastic lesions to neoplastic stages.

## GEMMs of Kras activation using gastric cell type-specific drivers

The study of Kras in the gastric cancer field was continued by multiple groups utilizing gastric cell type-specific driver mouse alleles, especially the genes specifically expressed in zymogen granule-secreting chief cells or proliferating-isthmal progenitor cells. The first study was performed using the Mist1-CreERT2 mouse allele to induce Kras activation in chief cells^11^. Constitutive Kras activation in chief cells led to pyloric metaplasia development only 1 month after active Kras expression. The pyloric metaplasia glands then progressed to intestinal metaplasia (IM) by 3 months and progressed to invasive glands at 4 months. Additionally, various metaplastic cell lineages in the glands were observed, including spasmolytic polypeptide-expressing metaplasia (SPEM) lineages produced by chief cell transdifferentiation and TFF3-expressing intestinal-type metaplastic cells. Gastric chief cells also secrete pepsinogen C (Pgc), a member of the aspartic protease family^85^. The Ito group generated a Pgc-mCherry-IRES-CreERT2 knock-in mouse allele (Pgc-CreERT2) using the Pgc gene locus^30^. The Pgc transcriptional activity was predominantly observed in chief cells but also in some of the mucus neck cells and isthmal progenitor cells. While Pgc-CreERT2;LSL-Kras^G12D^ mice showed pyloric metaplasia development only in the stomach corpus, mice with Apc and Trp53 deletion in addition to Kras activation developed invasive and metastatic carcinoma.

Several driver mouse alleles have also been generated to induce Kras activation in proliferating-isthmal progenitor cells using the eR1, Iqgap3, or Lrig1 gene locus^28,36,86^. These GEMMs developed a pyloric gland phenotype with a predominant expansion of the foveolar compartment rather than pyloric metaplasia with chief cell transdifferentiation, which is required for SPEM cell production.

## GEMMs of Kras induction using other gastric cell-type driver mouse alleles

Other investigators have developed GEMM models of gastric cancer that allow Kras activation in other gastric cell types. Kras activation in pit cells and progenitor cells predominantly through Tff1-Cre drivers led to the development of gastric atrophy and foveolar hyperplasia^87^. Interestingly, Kras^G12D^ expression combined with Cdh1 and Trp53 deletion in cells expressing the Atp4b gene gave rise to both intestinal- and diffuse-type tumors^88^. Additional driver mouse alleles, not specific for either stomach tissue or gastric cell types, have also been utilized to study gastric carcinogenesis. A gastric epithelium-specific CreERT2 mouse allele using the Anxa10 gene promoter (Anxa10-CreERT2)^29^, which is transcriptionally active throughout the stomach, showed tamoxifen-induced Cre recombination in all gastric cell types to model molecular subtypes of human gastric cancer, such as the CIN and GS subtypes^43^. In the CIN subtype, mutations in the Trp53, Kras, and transforming growth factor (TGF)-β pathways are frequently observed^43^. Anxa10-CreERT2 mice with Kras activation, a mutant form of Trp53 (Trp53R172H), and Smad4 deletion developed invasive intestinal-type gastric adenocarcinomas that metastasized to the liver and lung. CDH1 mutations are frequently observed in the diffuse GS subtype^43,89^. Notably, the Anxa10-CreERT2 mice with Cdh1 and Smad4 deletion, simultaneously with Kras activation, developed poorly differentiated tumors with diffuse-type gastric cancer morphology, which was histologically characterized by signet ring cells. In contrast, the Anxa10-CreERT2 mice with Cdh1 and Apc deletion along with Kras activation developed only serrated adenomatous gastric cancer. In addition, the Barker group recently generated a mouse model for gastric cancer using the Claudin18-CreERT2 driver mouse allele to achieve conditional mutations selective to both pyloric and corpus region of gastric epithelia^31^. Claudin18-CreERT2 mice with Apc and Trp53 deletion as well as Kras activation developed tumors that displayed histology similar to human advanced gastric cancers with distant metastases^31^.

## Conclusion and future perspectives

**Fig. 1:**
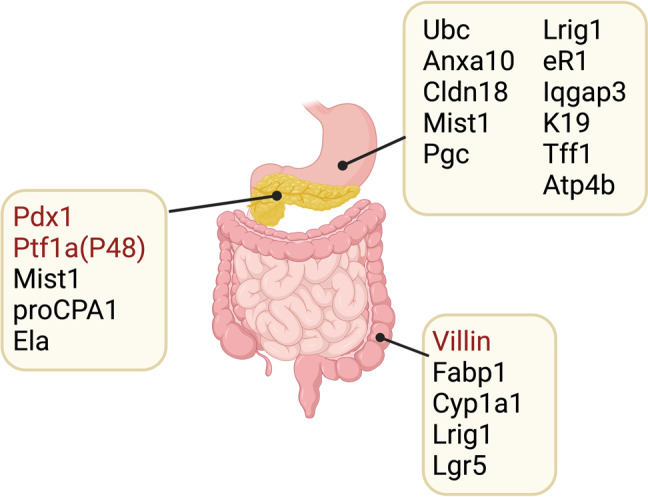
Driver genes used to express Kras. Numerous driver mouse alleles have been used to induce Kras activation in gastrointestinal organs, including the stomach, pancreas, and colon. While several key driver genes, such as Pdx1 and Ptf1a (P48) in the pancreas and Villin in the colon, have been commonly utilized, no driver mouse models that can faithfully recapitulate the carcinogenic process are available in the gastric cancer field yet. The image was created with BioRender.com.

Numerous GEMMs have been generated for GI tract cancer research, especially for gastric cancer research, and used to study GI epithelial cell carcinogenesis (Fig. 1). Many of these GEMMs have also been utilized as in vivo preclinical models for identifying therapeutic targets and examining drug effects. While there are several major driver mouse alleles for studying carcinogenesis in the pancreatic and colorectal cancer fields, there are still no mouse models of gastric cancer that faithfully recapitulate the full spectrum of the Correa pathway, undergoing mucosal changes from a normal mucosa to gastritis, metaplasia, dysplasia, and adenocarcinoma. This might be due to a lack of knowledge of a true cell of origin of gastric adenocarcinoma. Moreover, it is not clear whether Kras activation alone can lead to the full spectrum of gastric carcinogenesis, and other oncogenic gene activations are necessary for the full process or at least for the critical transition steps, preneoplastic metaplasia progression to neoplastic dysplasia or dysplasia evolution to adenocarcinoma. Notably, precancerous metaplasia, present not only in the stomach but also in the pancreas and colon, contains a common metaplastic cell population with features similar to SPEM cells^90,91^. This suggests that the metaplasia development induced by Kras activation is a generalized process that can at minimum lead to the initial step of carcinogenesis in GI organs. Therefore, defining the origin cell population of gastric cancer and establishing a novel driver mouse allele using gene loci specifically expressed in the origin cells would be critical for future gastric cancer research and for a better understanding of the roles of Kras activation during carcinogenesis.
